# Magnetic phase diagram of K_2_Cr_8_O_16_ clarified by high-pressure muon spin spectroscopy

**DOI:** 10.1038/s41598-018-37844-5

**Published:** 2019-02-04

**Authors:** Ola Kenji Forslund, Daniel Andreica, Yasmine Sassa, Hiroshi Nozaki, Izumi Umegaki, Elisabetta Nocerino, Viktor Jonsson, Oscar Tjernberg, Zurab Guguchia, Zurab Shermadini, Rustem Khasanov, Masahiko Isobe, Hidenori Takagi, Yutaka Ueda, Jun Sugiyama, Martin Månsson

**Affiliations:** 1 Department of Applied Physics, KTH Royal Institute of Technology, Electrum 229, SE-16440 Stockholm, Kista Sweden; 2 Faculty of Physics, Babes-Bolyai University, 400084 Cluj-Napoca, Romania; 3 Department of Physics & Astronomy, Uppsala University, SE-75121 Uppsala, Sweden; 4 Toyota Central Research and Development Laboratories, Inc., 41-1 Yokomichi, Nagakute, Aichi 480-1192 Japan; 5 Laboratory for Muon Spin Spectroscopy, Paul Scherrer Institute, CH-5232 Villigen, PSI Switzerland; 6 Max Planck Institute for Solid State Research, Heisenbergstraße 1, 70569 Stuttgart, Germany; 7 Toyota Physical and Chemical Research Institute, 41-1 Yokomichi, Nagakute, Aichi 480-1192 Japan

## Abstract

The K_2_Cr_8_O_16_ compound belongs to a series of quasi-1D compounds with intriguing magnetic properties that are stabilized through a high-pressure synthesis technique. In this study, a muon spin rotation, relaxation and resonance (*μ*^+^SR) technique is used to investigate the pressure dependent magnetic properties up to 25 kbar. *μ*^+^SR allows for measurements in true zero applied field and hereby access the true intrinsic material properties. As a result, a refined temperature/pressure phase diagram is presented revealing a novel low temperature/high pressure (*p*_C1_ = 21 kbar) transition from a ferromagnetic insulating to a high-pressure antiferromagnetic insulator. Finally, the current study also indicates the possible presence of a quantum critical point at *p*_C2_ ~ 33 kbar where the magnetic order in K_2_Cr_8_O_16_ is expected to be fully suppressed even at T = 0 K.

## Introduction

**Figure 1:**
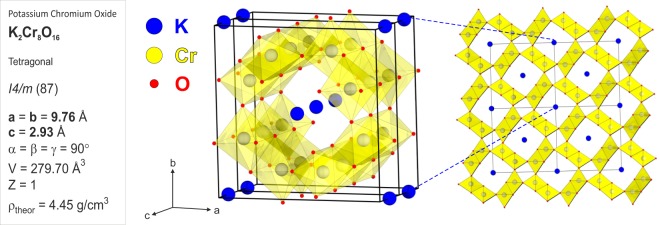
The crystallographic data and structure of K_2_Cr_8_O_16_ showing the tetragonal structure belonging to the *I*4/*m* space group with *a* = *b* = 9.76 Å and *c* = 2.93 Å. The middle panel shows two unit cells stacked along the *c*-axis, revealing the quasi-one-dimensional (Q1D) tunnel structure constructed from the Cr_2_O_4_ framework and K^+^ cation occupying the tunnel sites. The right panel is obtained if several unit cells are tiled within the ab-plane. Solid black lines indicate the unit cell, blue spheres represent the K ions, while the red and grey ones are the O and Cr ions, respectively.

Low dimensional magnets represent model materials, in which an intertwinning between electronic degrees of freedom leads to strongly correlated ground states^1–3^. Experimental realizations feature an enhanced interplay between quantum and thermal fluctuations, which can be tuned by several parameters, such as applied pressure/field or chemical substitution. In recent years, much attention has been paid to investigate the magnetic ground states of hollandite type materials^4–9^. These materials, with a general chemical formula *A*_*x*_*M*_8_O_16_, have a common quasi-one-dimensional (Q1D) tunnel structure composed from the *M*_2_O_4_ framework formed by the zigzag chains of edge-shared *M*O_6_ octahedra and an *A* cation at the tunnel sites, as shown in Fig. 1. In such systems, the formation of the magnetic ground state originates from a dominant intra-chain interaction together with a competition between the nearest and next nearest neighbor interactions^4,6^. In addition to their fundamental scientific interests, materials with similar open Q1D tunnel structures could potentially be used in future battery applications, where diffusion of the *A* cations is the main interest^10–12^.

One of the hollandite materials that has attracted a lot of attention for both theoretical and experimental studies is K_2_Cr_8_O_16_^13–26^. This compound has a tetragonal structure, space group *I*4/*m*^27^, and displays a rare mixed valence state, 2Cr^3+^ + 6Cr^4+^. While Cr^3+^ is considered to be very stable, only a few compounds display the Cr^4+^ state since it is only stabilized under extreme conditions^21^. This requires the synthesis and growth of the compound to be performed under high pressures (~6.7 GPa) resulting in very limited sample size/amount (100–500 mg), which clearly limits the range of experimental methods available to study its intrinsic physical properties. Furthermore, this compound displays an unexpected metal-insulator transition (MIT) at *T*_MIT_ = 95 K^14^, with the phases above and below the MIT temperature being ferromagnetic (FM). While the FM phase appearing below *T*_C_ = 180 K is well understood through the double exchange mechanism of itinerant *d*-electrons^14^, the microscopic mechanism behind the MIT has been a great puzzle for many physicists. Early X-ray diffraction measurements indicated no significant structural changes close to *T*_MIT_ and purely electronic/magnetic orgin was concluded^14^, *e*.*g*. the double exchange mechanism^28^. However, double exchange interaction usually implies metallicity and several theoretical studies were initiated^13,15,16,20^. First-principles electronic structure calculations revealed nesting of the Fermi surface and a charge/spin density wave was proposed^13,15^. Further calculations predicted charge ordering on two Cr sites, initiated by structural distortions^16^. This was confirmed by synchrotron X-ray diffraction experiments revealing a structural distortion from a tetragonal to a monoclinic phase at the MIT^17,18^. However, these studies combined with electronic structure calculations did not detect any charge ordering. Therefore, Peierls instability in the Q1D tunnels formed by four coupled Cr_2_O_4_ chains was instead suggested, with the double exchange interaction as the origin for the ferromagnetism, both above and below MIT^17,20^. Moreover, a previous muon spin rotation, relaxation and resonance (*μ*^+^SR) study^19^ and a recent photoemission study^24^ also support such Peierls transition scenario.

Previous bulk magnetic susceptibility studies of K_2_Cr_8_O_16_ reported a Curie-Weiss type behavior with a Curie temperature *T*_C_ = 180 K^14^, however, such data was obtained in a relatively high magnetic field (*B* = 0.1 T). It should be emphasized that the *μ*^+^SR technique allows for investigations at very low or even zero applied magnetic fields, meaning that the true intrinsic nature of the material can be acquired. In fact, previous *μ*^+^SR measurement revealed *T*_C_ ≃ 168 K^19^, emphasizing the significance of measuring in a magnetic field free environment.

So far, studies of the pressure dependency of the physical properties for this compound have been fairly uncommon due to limited sample volume and, consequently, the magnetic ground state of K_2_Cr_8_O_16_ at low temperatures and high pressures is still unsettled. For this purpose, the *μ*^+^SR method is powerful since the muon is a very sensitive magnetic probe and the method allows for measurements in low or even zero applied fields as well as low temperatures and high pressures. Here we present the first study of the true intrinsic magnetic ground state of K_2_Cr_8_O_16_ under high hydrostatic pressures using the *μ*^+^SR technique. Both zero-field (ZF) and weak-transverse field (wTF) field configurations were used and provided crucial information for clarifying and revealing a new magnetic phase diagram for this complex compound.

## Results

This section is divided into three subsections: *Zero-field (ZF)*, *Weak-transverse field (wTF)* and *The Phase diagram*. The first two explains the experimental data, analysis and the results obtained. The last subsection summarizes the obtained result in form of a phase diagram.

### Zero-field (ZF)

**Figure 2:**
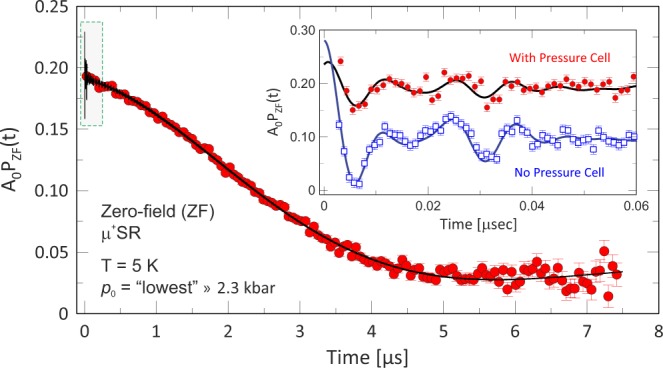
The Zero-field (ZF) muon spin rotation and relaxation (*μ*^+^SR) time spectrum at *T* = 5 K at the lowest applied pressure (*p*_0_ = 2.3 kbar). The longer time domain mainly displays a Kubo-Toyabe behavior with the main signal coming from the muons stopping in the non-magnetic pressure cell. The inset shows the shorter time domain where the muon spin precession, i.e. magnetic order in the sample, is clearly seen. Data from the current study (*p*_0_ = 2.3 kbar) are displayed as solid red circles and the result from the previous ambient pressure study^19^ as open blue squares. The solid lines are the fits of the respective ZF functions [Eq. (1)] to the data. The spectra are plotted in their absolute asymmetries and this study has a smaller oscillatory amplitude due to an elevated background component from the pressure cell.

The sample was measured under zero-field (ZF) at *T* = 5 K for several pressures ranging from *p*_0_ = 2.3 kbar to *p* = 25.0 kbar. The ZF time spectrum at the lowest applied pressure (*p*_0_ = 2.3 kbar) at *T* = 5 K is shown in Fig. 2. Analysis of the t ≥ 0.1 *μ*s time domain reveals a Kubo-Toyabe (KT) function, mainly coming from the pressure cell. The muon spin rotation frequencies, resulting from the Larmor precession of the muon spin around the internal fields of the magnetically ordered sample, can be obtained by analyzing the shorter time domain, t < 0.1 *μ*s as illustrated by the inset of Fig. 2. For reference, the result of the previous ambient pressure *μ*^+^SR^19^ experiment is shown in the same figure. Note that the spectra have not been shifted in the figure, indicating that the current study has a higher background asymmetry due to the pressure cell.

**Figure 3:**
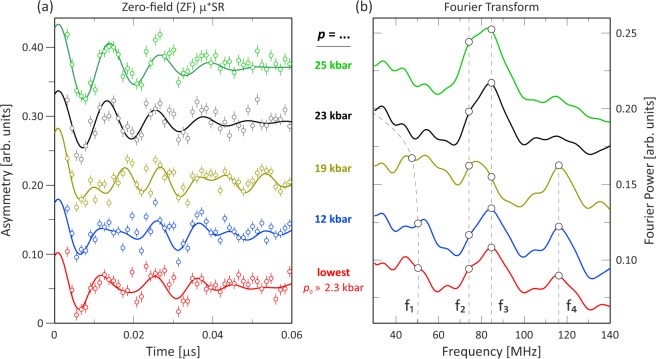
(**a**) Zero-field (ZF) time spectra at *T* = 5 K for each measured pressure and (**b**) the corresponding power Fourier transforms. The spectra have been shifted vertically for clarity of display. In (**a**), the respective fits using Eq. (1) are indicated by solid lines. In (**b**), the bullet points and dashed lines are guides to the eye.

Figure 3(a,b) display the ZF time spectra and their respective power Fourier transforms, recorded at *T* = 5 K for different applied pressures. The Fourier transform of the time spectrum at the lowest pressure (*p*_0_ = 2.3 kbar) indicates the presence of four major peaks, *f*_1_, *f*_2_, *f*_3_ and *f*_4_. Consequently, the time spectrum was accurately fitted using four oscillating signals, one non-oscillatory relaxing signal and one static gaussian Kubo-Toyabe signal:

1 $${A}_{0}\,{P}_{{\rm{ZF}}}(t)=\sum _{n=1}^{4}\,{A}_{n}^{{\rm{ZF}}}\,\cos ({\omega }_{n}t+{\varphi }_{n})\exp (\,-\,{\lambda }_{n}^{{\rm{ZF}}}t)+{A}_{{\rm{Tail}}}\exp (\,-\,{\lambda }_{{\rm{Tail}}}t)+{A}_{{\rm{KT}}}{G}^{{\rm{SGKT}}}({\rm{\Delta }},t),$$

where *A*_0_ is the initial asymmetry, *P*_ZF_(*t*) is the muon spin polarization function, *A*_n_, *ω*_n_, *ϕ*_n_, *λ*_n_ are the associated asymmetries, muon spin rotation frequencies, initial phases, and exponential depolarization rates, respectively, and *G*^*SGKT*^ represents a static gaussian Kubo-Toyabe function. Here, *A*_KT_ is the background asymmetry from the pressure cell while ${\sum }_{n=1}^{4}\,{A}_{{\rm{n}}}^{{\rm{Z}}F}$ and *A*_Tail_ represent the sample’s asymmetry. In a powder/multidomain sample, 2/3 of the muons precess around magnetic fields perpendicular to their initial polarization (the oscillation terms) while 1/3 of the muons sense a local magnetic field parallel to their initial polarization and do not precess (the non-oscillating tail). This illustrates one of the powers of *μ*^+^SR with the possibility to extract the magnetic volume fractions through the absolute values of the individual asymmetry components.

The following frequencies were obtained at the lowest pressure using the global fit procedure described in the next paragraph: *f*_1_ = 51.6(2), *f*_2_ = 76.04(3), *f*_3_ = 85.29(2) and *f*_4_ = 114.6(4) MHz where *f*_n_ = *ω*_n_/2*π*. Note that the obtained frequencies for lowest pressure are very similar to those from the previous *μ*^+^SR study^19^ at ambient pressure. There is a slight difference in the lowest frequency that could be related to the fact that this frequency has a very broad field distribution and will be more difficult to fit accurately, especially with the increased background from the pressure cell.

In Fig. 3(b), a clear pressure dependence is observed for the number of frequencies in the Fourier transform by comparing low pressure and the high pressure results. Both *f*_2_ and *f*_3_ are virtually constant throughout the entire pressure range, while *f*_1_ and *f*_4_ are strongly suppressed and finally vanish completely for the higher pressures *p* > 19.1 kbar. A closer look at the time spectra [Fig. 3(a)] highlights that the *μ*^+^SR spectra recorded at *p* = 23.0 and 25.0 kbar clearly contain less frequency components than those recorded at lower pressure. In addition, it was determined from individual fits, using Eq. (1), that *A*_Tail_ and *λ*_Tail_ were pressure independent up to *p* = 19.1 kbar and *p* = 25.0 kbar, respectively. As expected, the fitted value of *A*_Tail_ is about 1/3 of the sample’s full asymmetry. The maximum sample asymmetry for each pressure was determined from the wTF asymmetry well above the magnetic transition temperature, as detailed in the next subsection and Fig. 6(a). Combining the results of the individual fits with the Fourier transforms shown in Fig. 3(b) it is concluded that: (i) the frequencies (*f*_2_ and *f*_3_) are practically pressure independent and (ii) ${\lambda }_{{\rm{T}}ail} \sim 0$ *μs*^−1^, which indicates that the magnetic ordering is static for the whole pressure range. Therefore, the spectra at 5 K were fitted in a global fit by keeping *f*_2_ and *f*_3_ as common parameters for all pressures, while having *f*_4_ as a common parameter up to *p* = 19.1 kbar and fixing the *λ*_Tail_ = 0 *μs*^−1^. Finally, using a common but free phase (*ϕ*_1_ = *ϕ*_2_ = *ϕ*_3_ = *ϕ*_4_) for all pressures gave virtually the same fitting results (in terms of frequencies, depolarization rates, etc.) as when fixing the phase to zero. However, leaving the phase a free fitting parameter yielded a slightly better *χ*^2^, which reflects the expected complex field distribution in a sample with multiple muon sites and/or local field contributions^29^. Note that a *ϕ*_n_ = 0 implies the formation of a commensurate magnetic order in K_2_Cr_8_O_16_ up to the highest pressure measured.

**Figure 4:**
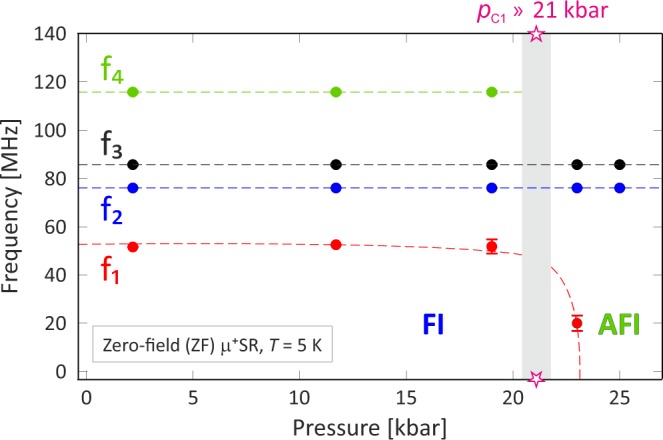
The four muon spin rotation frequencies as a function of pressure. Above *p* = 19.1 kbar, both *f*_1_ and *f*_4_ disappears and only *f*_2_ and *f*_3_ are remaining pressure independent. A phase transition from ferromagnetic insulator (FI) to antiferromagnetic insulator (AFI) occurs at the critical pressure (*p*_C1_ ≈ 21(2) kbar), indicated by the grey area.

The four muon spin rotation frequencies (*f*_1_ − *f*_4_) obtained through the global fit as a function of pressure are illustrated in Fig. 4. Between *p* = 19.1 kbar and *p* = 23.0 kbar, two of the four frequencies disappear and only f_2_ and f_3_ are remaining. This behavior clearly reveals the occurrence of a magnetic phase transition at *p*_C1_ ≈ 21(2) kbar, which is also apparent from Fig. 3.

### Weak-transverse field (wTF)

**Figure 5:**
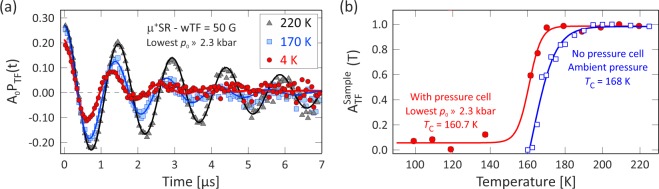
(**a**) Weak-transverse field (wTF = 5 mT) *μ*^+^SR time spectra at the lowest pressure measured (*p*_0_ = 2.3 kbar) as a function of temperature and the corresponding fits to Eq. (2). (**b**) Background (pressure cell) subtracted and normalized asymmetry plot as a function of the temperature, where the filled red circles are data from this study and the open blue squares are from the previous *μ*^+^SR experiments at ambient pressure^19^. The respective sigmoid fits are indicated as solid lines.

The magnetic properties of the sample were also studied by applying a weak-transverse field, wTF = 5 mT. Note that this field is significantly smaller than the internal ordered magnetic field at each muon site below the magnetic transition temperature. It is also much lower than the field applied in the previously published bulk measurements^14,25^ where *B* = 0.1 or 1 T were used. The time spectra of the wTF measurements at the lowest pressure for selected temperatures are illustrated in Fig. 5(a). The obtained spectra were fitted by using one relaxing oscillatory signal and one exponentially relaxing non-oscillatory signal:

2 $${A}_{0}\,{P}_{{\rm{TF}}}(t)={A}_{{\rm{TF}}}\,\cos ({\omega }_{{\rm{TF}}}t+{\varphi }_{{\rm{TF}}})\exp (\,-\,{\lambda }_{{\rm{TF}}}t)+{A}_{{\rm{S}}}\exp (\,-\,{\lambda }_{{\rm{S}}}t),$$

where *A*_0_ is the initial asymmetry, *P*_TF_(*t*) is the muon spin polarization function in wTF, *ω*_TF_ is the frequency from the applied wTF, *ϕ*_TF_ is the initial phase of the oscillatory signal, *λ*_TF_ and *λ*_S_ are the corresponding depolarization rates, while *A*_TF_ and *A*_S_ are the asymmetries (signal amplitudes) of the two components. Here, *A*_TF_ accounts for the signal from the p-cell and the paramagnetic fraction of the sample in the externally applied wTF and *A*_S_ is identical with A_Tail_ from Eq. (1).

The temperature dependence of the normalized sample component with the pressure cell contribution subtracted, ${A}_{{\rm{TF}}}^{{\rm{Sample}}}(T)=({A}_{{\rm{TF}}}-{A}_{{\rm{\min }}})/{A}_{{\rm{\max }}}$, at lowest pressure (*p*_0_ = 2.3 kbar) is shown in Fig. 5(b). Since ${A}_{{\rm{TF}}}^{{\rm{Sample}}}$ approximately corresponds to the paramagnetic volume fraction of the sample, the step-like ${A}_{{\rm{TF}}}^{{\rm{Sample}}}(T)$ curve indicates that the system changes from a low-temperature magnetically ordered state to a high-temperature paramagnetic state at around 160 K. More accurately, the transition temperature is extracted as the middle point of a sigmoid fit is *T*_C_ = 160.7(0.3) K. For reference, our previous *μ*^+^SR ambient pressure measurement^19^ is shown in the same figure with *T*_C_ = 168 K. The difference in transition temperatures is explained by difference in pressure. When the pressure cell with a sample and a pressure medium is sealed by hand, it is normal that a pressure of *p*_0_ ≈ 1–2 kbar is applied. A lower *T*_C_ (=160.7 K) is also consistent with the fact that the pressure is slightly higher, because the transition temperature decreases with pressure, as described below. Thus, the lowest applied pressure in the current study is estimated to *p*_0_ = 2.3 kbar by a linear extrapolation using the transition temperatures at *p* = 11.7 kbar and the ambient pressure data^19^. This is in line with what can be expected from closing and sealing the pressure cell using manual hand tools (as mentioned above).

**Figure 6:**
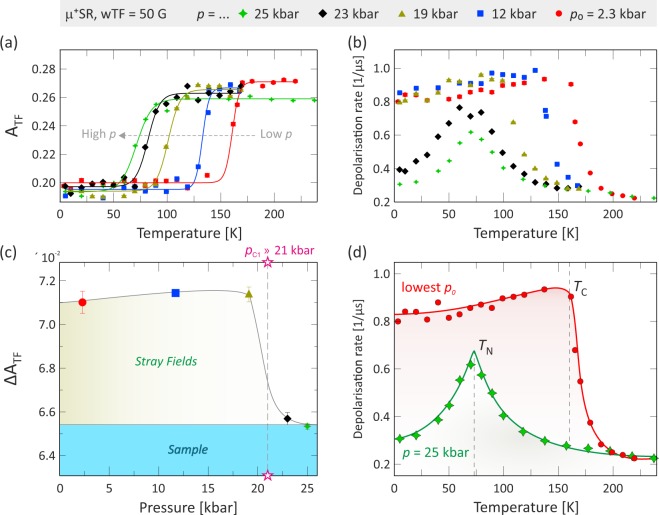
Fit results for the weak-transverse field (wTF) data using Eq. (2) namely, the temperature dependencies for all measured pressures of the: (**a**) wTF asymmetry [*A*_TF_(*T*)] and (**b**) wTF depolarization rate [*λ*_TF_(*T*)]. (**c**) The difference of A_TF_ above and below the magnetic transition temperature (Δ A_TF_) as a function of pressure. (**d**) Close-up view of *λ*_TF_(*T*) acquired at the lowest and highest pressures. These results demonstrate a drastic change in magnetic order due to the transition from a FM (*T*_C_ = 160.7 K at *p*_0_ = 2.3 kbar) phase to an antiferromagnetic (*T*_N_ = 71.2 K at *p* = 25.0 kbar) phase at *p*_C1_ = 21(2) kbar.

The temperature dependencies of *A*_TF_ and *λ*_TF_ for each applied pressure are obtained from fits of the corresponding *μ*^+^SR spectra using Eq. (2) and are displayed in Fig. 6. A magnetic phase transition is clearly visible from the drastic change in both *A*_TF_ and *λ*_TF_. The transition temperature is shifted towards lower temperatures [Fig. 6(a)] with increasing pressure. Interestingly, *λ*_TF_(*T*) [Fig. 6(b)] drastically changes its behavior above *p*_C1_ [Fig. 6(d)]. At lower pressures, *λ*_TF_(*T*) display an abrupt increase at *T*_C_ and then keeps a constant value at lower temperatures. Above *p*_C1_ ≈ 21 kbar, however, *λ*_TF_(*T*) shows a sharp cusp at the magnetic transition temperature and then rapidly decreases with decreasing temperature. Such drastic change in behavior suggests the presence of a pressure induced magnetic phase transition between *p* = 19.1 kbar and *p* = 23.0 kbar. This finding is discussed in greater detail in Sec. 3.

Looking closely at the TF asymmetries, a subtle difference can be discerned between the low and high pressure magnetic phases. As emphasized in Fig. 6(c), the low pressure asymmetries display a bigger change in comparison to the high pressured one when crossing the magnetic transition temperature. As discussed in Sec. 3, this is most likely related to a change in magnetic order of the sample, inducing weak stray fields in the neighboring pressure cell.

### The Phase diagram

**Figure 7:**
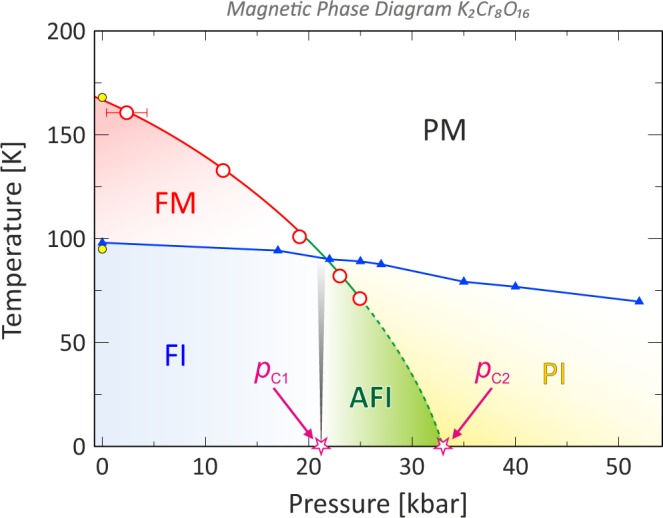
The novel detailed P-T phase diagram of K_2_Cr_8_O_16_. The red circles indicate the transition from a paramagnetic (PM) to a magnetically ordered state while the dashed line is an extrapolation. The faded black line is the phase transition boundary between the ferromagnetic insulating (FI) to the newly determined antiferromagnetic insulating (AFI) phase. *p*_C1_ ≈ 21(2) kbar and *p*_C2_ ≈ 33 kbar are the critical pressures identified by the current *μ*^+^SR experiment. The blue triangles/line indicate the metal-insulator transition (MIT) lines from previously published data^25^, which together with the *μ*^+^SR data uniquely determine the high-pressure and low-temperature phase (yellow region) as a paramagnetic insulator (PI).

Based on the results obtained from both ZF and wTF measurements, we have have constructed a novel and detailed magnetic phase-diagram for K_2_Cr_8_O_16_ (see Fig. 7). The pressure dependence of the MIT temperature^25^ is displayed in Fig. 7 as blue symbols/line. The transition temperature and the observed MIT from the previous ambient pressure *μ*^+^SR study^19^ are also included as indicated by the yellow circles. Note that this MIT temperature is in good agreement with the resistivity measurements.

The pressure dependence of the transition temperature, obtained from wTF *μ*^+^SR experiments, is shown as red circles in Fig. 7. The transition temperature is decreasing with higher pressure, in agreement with published bulk transport measurements^25^, however, the current *μ*^+^SR data yields slightly lower (~10 K) overall phase boundary. Since our *μ*^+^SR measurements are performed at zero or very low applied fields, the current results represent the true intrinsic magnetic properties of K_2_Cr_8_O_16_. Even though the maximum applied pressure is approximately 25 kbar^30,31^, the extrapolation of the red curve (dashed green line in Fig. 7) suggests the complete suppression of magnetic order in K_2_Cr_8_O_16_ at about ${p}_{{\rm{C}}2} \sim 33$ kbar, i.e. the existence of a quantum critical point. Here it should also be emphized that the current *μ*^+^SR data show the presence of a non-magnetic state just below the MIT for *p* = 23.0 kbar and *p* = 25.0 kbar. The high-pressure, *p* ≥ 33 kbar, and low-temperature phase is assigned as a paramagnetic insulator (PI). Such results also conclusively resolve the findings of ref.^25^ that reported this phase as either an antiferromagnetic insulator (AFI) or a PI.

The transition pressure (*p*_C1_ = 21(2) kbar) between the two magnetically ordered states at low temperatures is indicated in Fig. 7 with a vertical black line. This line is faded to indicate that it is extrapolated vertically from the ZF data acquired at *T* = 5 K up to where the *T*_C_ line (red circles/line) is crossing the blue MIT line. Consequently, ZF and wTF data are very consistent and additional evidences to further support why such phase is assigned as an AFI ordered phase is described in Sec. 3.

## Discussion

This section includes discussions regarding how the low-temperature phase between *p*_C1_ = 21(2) kbar and *p*_C2_ = 33 kbar is assigned to be AFI. Note that this phase was already firmly determined to be an insulating phase from previous resistivity study^25^. First of all, it is clear from both ZF and wTF measurements that this phase has to be magnetically ordered, i.e. not a PM phase. At ambient pressure and low temperatures, K_2_Cr_8_O_16_ enters into a FM phase. The internal field at each muon site in ZF can be expressed as^32^:

3 $${\overline{H}}_{\mu }={\overline{H}}_{\mathrm{Di}{\rm{p}}^{\prime} }({r}_{{\rm{\mu }}})+{\overline{H}}_{{\rm{Hyp}}}({r}_{{\rm{\mu }}})+{\overline{H}}_{{\rm{L}}},$$

where *H*_Dip′_ is the summation of dipolar fields within a Lorentz sphere, *H*_Hyp_ is the hyperfine contact field, r_μ_ is the distance from the magnetic ion to the muon site and *H*_L_ is the Lorentz field. For paramagnetic and antiferromagnetic materials, both *H*_Hyp_ and *H*_L_ are usually very small, essentially zero, hence, the dipole field will solely dominate the contribution to the muon spin rotation frequency in such a state. Looking at the pressure dependence of the current ZF *μ*^+^SR data [see Figs 3 and 4], it is clear that the number of frequencies, *f*_*n*_, in the *μ*^+^SR spectra decreases from four to two, when crossing *p*_C1_ ≈ 21(2) kbar. This behavior can be explained by either a magnetic transition, structural transition or a combination of both. A structural transition seems unlikely since f_2_ and f_3_ are independent of pressure and a change of muon sites would have dramatically changed the internal dipole field contributions. Instead, the changes in the number of frequencies can be explained by a magnetic transition. From the wTF results, we will show that this fact can be ascribed to a change from FI to AFI order.

**Figure 8:**
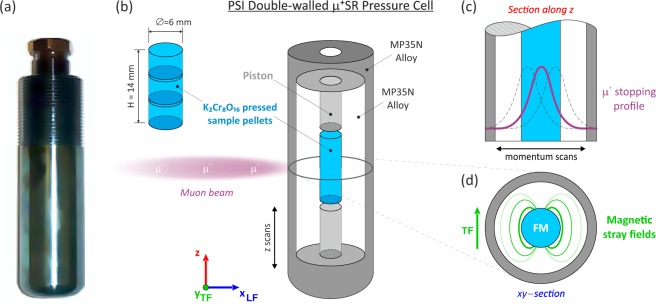
The double-walled pressure cell for *μ*^+^SR measurements at the GPD beamline. The direction of the applied field is marked as TF for transverse field in green and LF for longitudinal field in blue. (**a**) Photo of the pressure cell made of MP35N high-strength alloy. (**b**) Schematic view of the pressure cell with the stacked sample pellets and the vertical z-scans. (**c**) Schematic view of the muon momentum scans performed to maximize the samples signal (i.e. minimize background signal from the pressure cell). (**d**) Schematic view on how the sample in a ferromagnetic (FM) state induces stray fields with high field-distribution width inside the surrounding non-magnetic pressure cell.

In general, at a second order phase transition, the *λ*_TF_(*T*) curve exhibits a sharp maximum at the phase transition temperature^33–36^ with a typical critical behavior related to the formation of magnetic order. This is also the case for K_2_Cr_8_O_16_ in our previous ambient pressure *μ*^+^SR study^19^ where *λ*_TF_(*T*) displays a sharp cusp at *T*_C_ but have values close to zero below the transition. This behavior originates from the fact that the internal magnetic field, caused by the magnetic order, is considerably greater than the applied wTF. For the current high-pressure study, the situation is a bit more complex. Looking carefully at *λ*_TF_(*T*) in Fig. 6(d), for *p* ≤ 19 kbar, it is clear that *λ*_TF_ is temperature independent even below the transition temperature. Such situation drastically changes at *p* ≥ 23 kbar (*p*_C1_ ≈ 21(2) kbar) where the *λ*_TF_(*T*) curve exhibits a typical critical behavior with a maximum at the phase transition and then quickly decreases again as temperature is lowered. In order to explain this, we should note that for a high-pressure experiment some muons (~26%) stop in the sample and the remaining muon fraction (~74%) stop in the non-magnetic pressure cell. At low pressures, K_2_Cr_8_O_16_ is known to be in a ferromagnetic state, which induces a magnetization by the externally applied field. This would naturally cause a weak stray-field from the sample into the surrounding non-magnetic pressure cell [as schematically illustrated below in Fig. 8(d)]. Such stray-field will provide a very broad field-distribution width in the sample and cell below *T*_C_, leading to large *λ*_TF_. However, when passing *p*_C1_ ≈ 21(2) kbar, *λ*_TF_ decreases with decreasing temperature below the transition temperature and recovers to a value expected for a MP35N pressure cell. This indicates absence of stray-fields inside the pressure cell meaning the high-pressure magnetic phase is no longer FI but, as explained below, AFI [*T*_N_ = 71.2 K at *p* = 25.0 kbar], i.e. a phase without induced magnetization under applied magnetic field.

Having the wTF depolarization rate in mind, also the wTF asymmetry (*A*_TF_) strengthen the case for an AFI phase above *p*_C1_ ~ 21 kbar. As shown in Fig. 6(c), the sample’s asymmetry, i.e. the difference of *A*_TF_ above and below the magnetic transition temperature (Δ*A*_TF_), varies with pressure. That is, at low pressures with *p* ≤ 19 kbar, Δ*A*_TF_ ~ 0.07, while Δ*A*_TF_ ~ 0.06 at *p* ≥ 23 kbar. This could be explained in a similar manner as in the case of *λ*_TF_. When K_2_Cr_8_O_16_ is in the FI state, stray-fields, larger than the wTF = 5 mT, are induced in the adjacent pressure cell. As a consequence, wTF *μ*^+^SR experiments provide a “false” increase of the sample’s asymmetry, i.e. a decrease of the *A*_TF_, below *T*_C_. On the contrary, in the AFI phase above *p*_C1_ ≈ 21(2) kbar, the whole pressure cell returns into an entirely non-magnetic state due to the absence of the stray-fields. Therefore, muons stopping in the pressure cell do no longer contribute to a loss of *A*_TF_ below the transition temperature.

It has also been proposed that the K_2_Cr_8_O_16_ compound at high pressures and low temperatures enters into a more complex magnetic structure^25^, *e*.*g*. an island structure or perhaps a spiral spin order. An island scenario is, however, clearly excluded, because the *μ*^+^SR data does not show the expected separation of different magnetic volume fractions. An incommensurate spiral order is also not supported since the ZF-*μ*^+^SR spectrum is accurately fitted with *ϕ*_n_ ≈ 0°, indicating the presence of commensurate magnetic order. A typical incommensurate magnetic ordering is characterized by a broad field distribution limited by two well separated fields, B_max_ and B_min_. The transition from a ferromagnetic to an incommensurate magnetic structure would imply an abrupt increase of the depolarization rates, which is not the case in our data (see Fig. 3(b)). Such rapidly depolarizing oscillation should also be fitted by the product of zeroth-order Bessel function of the first kind [*J*_0_(*t*)] and a cosine function, rather than an single exponentially relaxing cosine function^37^. For K_2_Cr_8_O_16_, however, both below and above *p*_C1_ ≈ 21(2) kbar, fits using any combination of *J*_0_(*t*) yields very poor results. Based on the fact that two frequencies remain the same below and above *p*_C1_, with similar depolarization rates and the stray field disappear at low temperatures, above *p*_C1_, the most likely simple scenario is that the sample undergoes a FI to AFI transition. Nevertheless, commensurate spiral order could still be a candidate for the magnetic structure at high pressures and low temperatures. In order to gain further insights into the subtle details of the spin order, neutron diffraction measurements under high pressure and low temperature are needed. Unfortunately, such experiments are challenging due to the limited available sample volume from the high-pressure synthesis. Another route to more accurately determine the magnetic ground state would be a combination of the current muon data with detailed structural analysis from high-resolution x-ray diffraction (XRD). Information on subtle structural changes with pressure could allow us to deduce possible changes in muon stopping site(s) with changes in the individual Larmor frequencies. Such information could give a possibility to determine a more detailed spin structure from *μ*^+^SR^29,38^, particularly in combination with first principles calculations for predicting the muon site(s).

In summary, the present ZF and wTF *μ*^+^SR results reveal that the low-temperature magnetic phase at *p* ≥ *p*_C1_ ≈ 21 kbar is an AFI phase, although the previous bulk magnetization measurements^25^ suggested either a ferro- or ferrimagnetic phase. However, it should be noted that looking carefully at the data in Fig. 3(a) of ref.^25^ the magnetization curve display an evident decrease below the magnetic transition temperature for pressures above *p*_C1_. Such a decrease in magnetization with lowering of the temperature is a clear sign of an antiferromagnetic spin order. However, since the magnetization below the transition temperature did indeed not approach zero value, the phase was instead identified as ferrimagnetic rather than antiferromagnetic. Now having the present *μ*^+^SR data available, we are able to revisit and potentially re-evaluate the previous magnetization data^25^. There can be several reasons why the magnetization does not decrease to zero below the AFI transition temperature (*T*_N_). First of all, the sample is known to contain a small fraction (≲2%) of a highly ferromagnetic CrO_2_^28,39–41^ impurity phase, which has shown to retain ferromagnetic order also at higher pressures^42^. Such impurity phase will clearly cause the entire sample to have a ferromagnetic background on top of the AFI signal, which in a bulk magnetic measurement cannot be separated from a ferrimagnetic state. In a muon measurement this is not a problem since one of the powers of *μ*^+^SR is the capability to separate individual magnetic volume fractions and hereby easily distinguish between a ferrimagnetic phase vs. an AFI + FM impurities. The current *μ*^+^SR data strongly supports the latter. Note that a strong ferrimagnetic state would cause similar stray fields in the pressure cell as the ferromagnetic state and can therefore be excluded. However, a nearly compensated ferrimagnetic phase (i.e. almost AFI) would yield a similar *λ*_TF_(*T*) temperature dependence [Fig. 6(d)]. Therefore, such phase should not be completely excluded. Nevertheless, the ZF data shown in Fig. 4 suggest that AFI phase is more likely compared to a nearly compensated ferrimagnetic phase. In addition to the impurity scenario, it should once again be emphasized that the magnetization data^25^ was acquired under a rather high externally applied magnetic field (*B* = 0.1 and 1.0 T), while our current *μ*^+^SR data was recorded under very low (*B* = 5 mT) or even zero applied field. Finally, it should also be noted that *μ*^+^SR and bulk magnetization covers very different times and length scales.

In conclusion, the current *μ*^+^SR investigations under high pressure have yielded a novel insight on the magnetic order and transitions in K_2_Cr_8_O_16_. A new and more detailed *P* − *T* phase diagram is presented where two phase transitions at *p*_C1_ ≈ 21 kbar and *p*_C2_ ≈ 33 kbar are found for *T* = 5 K. Combining the ZF and wTF muon data, the first transition is uniquely assigned as a low-pressure ferromagnetic insulator phase to a medium-pressure antiferromagnetic insulator phase (FMI-to-AFI) and the second one as a medium-pressure antiferromagnetic insulator phase to a high-pressure paramagnetic insulator phase (AFI-to-PI). The latter would then indicate the presence of a quantum critical point in K_2_Cr_8_O_16_. This demonstrates a unique power of the *μ*^+^SR technique to provide fundamental information on the magnetic ground state in complex compounds, even in zero applied field.

## Methods

The sample was prepared under high pressure and high-temperature conditions with a Walker-type multianvil press at Max Planck Institute for Solid State Research, by a solid-state reaction of a mixture of K_2_Cr_2_O_7_:Cr_2_O_3_ = 1:3 in a sealed platinum-foil capsule under 7 GPa at 1100 °C for 1 h. The amount of the obtained sample was a few hundred milligrams in each synthesis batch. The characterization of the sample is reported in refs^14,18^. The sample used for the current high-pressure *μ*^+^SR measurements was characterized using x-ray diffraction (XRD), magnetic susceptibility and ambient pressure *μ*^+^SR in order to confirm the high sample quality.

Three pressed pellets of the powder samples were stacked (6 mm diameter and 14 mm total height) for each series of measurements. The sample pellet was inserted into a piston-cylinder clamp cell made of MP35 alloy^30^ [see also Fig. 8(a,b)]. Daphne oil was used as a pressure medium to apply hydrostatic pressure up to 25 kbar and a ^3^He cryostat was used to achieve temperatures down to *T*_Base_ = 0.3 K. The pressure inside the cell was determined by *in situ* AC susceptibility measurements for the superconducting transition temperature of an indium wire located at the bottom of the cell. Zero-field (ZF) and weak-transverse field (wTF = 50 G) *μ*^+^SR measurements were performed at the GPD instrument^30,31^ on the *μ*E1 beamline of the Paul Scherrer Institute (PSI) in Switzerland. Vertical sample position scans [adjustable by few mm, see Fig. 8(b)] and muon momentum scan (adjustable by few percent, Fig. 8(c)]) were performed before each initial sample setup in order to maximize the cross section between the muon beam and the sample (i.e. minimize the background asymmetry from the pressure cell). More detailed information of the high-pressure *μ*^+^SR experimental setup can be found in refs^30,31^. Finally, the software package musrfit^43^ was used to analyze the *μ*^+^SR data.
